# First, do no harm. Ethical and legal issues of artificial intelligence and machine learning in veterinary radiology and radiation oncology

**DOI:** 10.1111/vru.13171

**Published:** 2022-12-13

**Authors:** Eli B. Cohen, Ira K. Gordon

**Affiliations:** ^1^ Department of Molecular and Biomedical Sciences North Carolina State University College of Veterinary Medicine Cary North Carolina USA; ^2^ The Oncology Service by United Veterinary Care Knoxville Tennessee USA

**Keywords:** AI, artificial intelligence, ethics, machine learning, medicolegal

## Abstract

Artificial Intelligence and machine learning are novel technologies that will change the way veterinary medicine is practiced. Exactly how this change will occur is yet to be determined, and, as is the nature with disruptive technologies, will be difficult to predict. Ushering in this new tool in a conscientious way will require knowledge of the terminology and types of AI as well as forward thinking regarding the ethical and legal implications within the profession. Developers as well as end users will need to consider the ethical and legal components alongside functional creation of algorithms in order to foster acceptance and adoption, and most importantly to prevent patient harm. There are key differences in deployment of these technologies in veterinary medicine relative to human healthcare, namely our ability to perform euthanasia, and the lack of regulatory validation to bring these technologies to market. These differences along with others create a much different landscape than AI use in human medicine, and necessitate proactive planning in order to prevent catastrophic outcomes, encourage development and adoption, and protect the profession from unnecessary liability. The authors offer that deploying these technologies prior to considering the larger ethical and legal implications and without stringent validation is putting the AI cart before the horse, and risks putting patients and the profession in harm's way.

## 1 INTRODUCTION

### 1.1 Background

In order for artificial intelligence (AI) to be trustfully adopted in veterinary medicine, it needs to be lawful, ethical, and robust. As veterinary medicine evolves with new technologies, veterinarians are charged with upholding and adhering to ethical conduct. This is particularly important in the realm of AI because technology innovators may be unfamiliar with, or insensitive to healthcare policy or medical and research ethics. Accordingly, it is the role of the veterinary community (specifically, veterinary radiology and radiation oncology as domain experts) to educate and ensure ethical adoption of AI in veterinary diagnostic imaging and radiation oncology.

### 1.2 Artificial intelligence in medicine

AI can be broadly defined as the design of computer systems to do things that would require intelligence if a human were to perform the same task.^1^ Including applications such as voice recognition, pattern recognition and identification, or complex automation. In radiology, AI most frequently refers to computerized image analysis and interpretation.

AI is distinct from image processing, which is a method to enhance an image or extract key image features. While both involve the use of a software algorithm, AI is often developed by machine learning (ML), applying an algorithm to large data sets with some knowledge associated with that data.^2^ The quality of AI predictions depends on the quality of training image data, knowledge about the training image data, inherent features of the algorithm itself, and consistency of correlations between imaging and biology. Basic algorithms may look at specific features of a CT scan (ie. Pre and post‐contrast HU, size, heterogeneity), but more complex problem‐solving algorithms may have hundreds of layers intended to mimic the neural networks of the human brain. These algorithms can ‘learn’ without human supervision, drawing from data that is unstructured and unlabeled.^3^

### 1.3 Relevant veterinary medical ethical principles to be applied to AI

The American Veterinary Medical Association (AVMA) outlines the principles of ethical conduct in the practice of veterinary medicine. Several of these principles are particularly relevant to the development and implementation of technologies such as AI in diagnostic imaging and are paraphrased below^4^:

- Veterinarians should be influenced only by the welfare of the patient, needs of the client, and safety of the public.
- Clinical care shall be provided under the terms of a veterinarian‐client‐patient relationship (VCPR).
- Veterinarians shall safeguard medical information within the confines of the law.
- Veterinarians must protect the privacy of clients and must not reveal confidences unless it becomes necessary to protect the health and welfare of other individuals or animals.
- Medical records are the property of the practice and the practice owner. Information within veterinary medical records is confidential. It must not be released except as required or allowed by law, or by consent of the owner of the patient
- Without express permission of the practice owner, it is unethical for a veterinarian to remove, copy, or use medical records for personal or professional gain.
- Veterinarians shall continue to study, apply, and advance scientific knowledge.

Veterinarians are the only professionals licensed to diagnose and treat diseases in animals. In addition to the ethical guidelines that veterinarians adhere to, laws and regulations define and limit the scope of when, where, and how veterinarians practice. These guidelines, laws, and regulations help ensure the safety of patients and the public, but AI had not yet emerged when they were conceived. This problem is not unique to medicine. Indeed, regulators are currently struggling to define how to ensure safety and responsibility for many AI tools ranging from self‐driving cars to facial recognition.^5^

### 1.4 Veterinary ethical principles applied to AI in diagnostic imaging and radiation therapy

In consideration of the ethical principles described above, AI should be adopted in veterinary diagnostic imaging and radiation therapy only when it improves the welfare/outcomes of the patients, needs of the client, and/or safety of the patient. It should be applied to clinical care under the terms of a VCPR.

The principle of ‘informed consent’ is critical to the clinical use of AI in medicine. It should not be assumed that pet owners understand what AI entails.^6^ AI providers should specifically describe how pet health data is obtained, stored, and used. A disclosure should be provided to pet owners stating what personal or personally identifiable information is shared, who has access to this data, and for what purposes. This includes whether the data or products created from their data will be sold or shared with outside parties. One ethical concern involving AI in diagnostic imaging is the ‘black box’ nature of many algorithms.^7^ Veterinarians may not be able to understand how or why an AI tool has made a certain determination or recommendation. Knowledge held within an algorithm that cannot be understood or shared with the medical community cannot be reasonably analyzed or reviewed. It may be subject to biases based on the population of previous inputs that might not be identifiable until severe errors or outcomes occur. Algorithms may be constantly learning, and without an understanding of their process, we cannot ensure accuracy and resultant patient safety over the course of its use. Veterinarians should disclose to pet owners when AI is a part of their pet's diagnosis, and their understanding of the diagnosis and the accuracy/reliability of that information. A veterinarian's ability to provide an accurate disclosure will depend on transparency from AI providers.

When a misdiagnosis or medical error occurs (such as inappropriate dose delivery in radiation oncology), a root cause analysis should be performed. This systematic process seeks to identify the cause of an adverse event, in order to prevent the same error in the future.^8^ If an AI system causes harm, we must be able to understand why. New procedures will be needed to analyze adverse AI outcomes.

## 2 ETHICAL ADOPTION OF AI

### 2.1 Guiding principles

As with any new and disruptive technology, AI has the potential to change how we practice veterinary medicine. This will happen in some ways we can predict, and likely in many ways we cannot. As we usher in this novel technology, it is incumbent on us as a profession to establish guiding principles to safeguard our animal patients, their human owners, and our veterinary colleagues. It is useful to remind ourselves of the veterinarian's oath:

“Being admitted to the profession of veterinary medicine, I solemnly swear to use my scientific knowledge and skills for the benefit of society through the protection of animal health and welfare, the prevention and relief of animal suffering, the conservation of animal resources, the promotion of public health, and the advancement of medical knowledge.”

I will practice my profession conscientiously, with dignity, and in keeping with the principles of veterinary medical ethics.

“I accept as a lifelong obligation the continual improvement of my professional knowledge and competence.^9^”

Within this oath that all veterinarians take prior to entering into practice are a few notable principles directly applicable to AI. We are charged to use our skills and knowledge to balance sometimes‐opposing needs of reducing animal suffering and improving welfare with the advancement of medical knowledge. Even without AI, we are well aware that these facets of our oath can sometimes be at odds. This is why the oath requires our actions and practice be guided by ethics, so that scientific advancements are not instituted blindly. In research, advancing medical knowledge sometimes creates suffering or reduction of health of an individual animal for the benefit of many animals. These decisions are made with appropriate review processes in place such as institutional animal care and use committees (I.A.C.U.C.), which serve to ensure that investigations are performed in such a way to limit animal numbers (and resultant suffering), have appropriate study design to address the question at hand, and create defensible downstream data which we as veterinarian's can employ to improve our practice. As we are also charged with lifelong learning and increasing our knowledge and competence, we must learn to embrace developing technologies while safeguarding our patients. Alongside the veterinarian's oath, we are guided by the chief principle of the Hippocratic oath, *primum non nocere*, or first, do no harm. In order to adhere to this tenet, we must have a strong understanding of the consequences of how we practice, and the effects of the technologies we employ. If we do not know or cannot assess the accuracy of an AI product, we have no way to gauge the potential harm of its use. It is therefore obligatory for us as veterinarians to ensure we know both the potential upsides (increased efficiency, increased accuracy, error reduction) and downsides (misdiagnosis, incomplete diagnosis, patient harm) of AI as we consider when and how it should be employed in veterinary practice. To quote Potter Stewart, “*Ethics is knowing the difference between what you have a right to do and what is right to do*.” In veterinary medicine we have the ability to perform euthanasia and the responsibility to determine when this is ethically indicated. When releasing a product or deciding upon using it in clinical decision‐making, we should consider what could be termed the “Euthanasia Principle”. Namely, is the decision or recommendation of an AI product defensible and validated to a sufficient degree so as to be ethical when that decision warrants euthanasia. It is incumbent on us as a profession, AI developers, and end‐users of this technology to inject a conscience and ethics in a product that inherently has none.

In the case of veterinary radiologists and radiation oncologists, the best way to have this level of understanding of AI is to be “in the loop” from development to deployment.^3^, ^10^ Direct involvement of a human domain expert serves to guide what parts of imaging and therapy practice would be best served by the addition of AI, aides in curation and assessment of the data sets to create those algorithms, and expert oversight and assessment of their performance. Our unique skillsets and knowledge should be leveraged to maximize AI, and AI used to magnify those skillsets, rather than seek to create human obsolescence. Our expertise lies beyond simply seeing a lesion or making a radiation target, but in our clinical decision making and synthesizing the totality of a case in conjunction with imaging in order to provide the best patient care. Narrow use AI (AI for a specific limited task) will be first to arrive, but general use AI (AI behaving as human intelligence) may also become a reality. Even for the latter, a human in the loop will remain necessary to provide oversight and conscience/ethics. Until an AI system is created and proven to be 100% accurate in all patients and settings (unlikely, but not impossible), a human in the loop is necessary to intervene when errors/failures occur (ideally prior to any patient detriment).

A recent charter endorsed by corporations such as Microsoft and IBM introduced the idea of “algor‐ethics,” with guiding principles of transparency, inclusion, accountability/responsibility, impartiality, reliability, security, and privacy.^11^ When considering AI use that augments or potentially replaces what veterinarians do, we must examine the concepts of standard of care (SOC) and best practice (BP). Patient care is a spectrum, but SOC can be broadly regarded as the minimum level of acceptable care.^12^ Legally, veterinary SOC is adapted from prior human cases, prescribed as, “the standard of care required of and practiced by the average reasonably prudent, competent veterinarian in the community.”^13^ This is the ordinary level of care, and does not carry the expectation that the average veterinarian will have the highest level of knowledge and skill. A specialist such as a veterinary radiologist or radiation oncologist is held to a higher standard, that of a competent member of their specialty.^14^ While BP (sometimes termed gold standard) is not currently feasible in every instance, it is reasonable that a radiologist interpreting images or a radiation oncologist planning and performing radiation therapy can be considered BP. If AI is to be employed in a way that augments or replaces specialist‐level practice as it is currently being marketed, it should be held to this higher BP standard. While current efforts are largely aimed at replacement of human expertise, it stands to reason that employing AI to extend and magnify human expertise should be the target for new BP.

### 2.2 Ethical development

Ethical AI development encompasses everything from data ethics on a granular level to the overall purpose of AI development (improving patient care, profit generation).^15^ Transparency of AI products, the companies producing them, the data used to create them, and the systems in place to assess performance, errors, and bias is what will ultimately either engender trust and adoption or mistrust and opposition/hesitancy. SOC in veterinary medicine is not currently radiologist interpretation of all imaging studies, but it is reasonable to posit that review of all imaging studies by a radiologist/domain expert would constitute BP. Radiologist workforce capabilities don't currently allow for this, but this is a reasonable goal for the profession to work towards. In regards to ethical AI development, it stands to reason that a radiologist (or radiation oncologist) in the loop would also constitute BP.^3^, ^9^, ^16^ BP is not only data set review by a domain expert (though this is an important consideration), but also direct involvement of the domain expert in the planning, development, deployment, and subsequent assessment of AI products. The absence of direct domain expert involvement in the development of a product seeking to supplant or augment a domain expert's role seems questionable at best from an ethical standpoint. A domain expert is best suited to determine what questions/applications should be addressed and in what order, provide guidance in data set choice and collation, assessment of performance and errors with safeguarding against the latter, and implementation of the product in a manner to increase access to BP medicine.^3^, ^15^

The “black box” nature of AI means that we as humans may not know (or be able to comprehend) how an AI product reaches a clinical decision, particularly one that employs deep learning.^16^, ^17^ This raises the question as to whether a veterinarian is justified to make a clinical decision (e.g. euthanize a patient) based on an algorithm output (e.g. pulmonary nodules identified on a thoracic radiograph indicating metastatic neoplasia) in which the clinician does not know how the algorithm reached the decision. AI can be developed so as to expose how the algorithm or neural network is working and generating an output. This transparency would provide key insight into errors and pitfalls, which could then be used to optimize the system. This directly applies to products that may identify individual diagnoses or imaging signals (as opposed to general AI). If we do not know how diagnoses are reached we inherently cannot understand when the AI could be incorrect when it encounters novel image sets. The “black box” concept could also be applied to the transparency of AI companies, including what validation data, performance data, and error monitoring they disclose (or is even available). Veterinary products do not fall under the same regulatory guidelines as human products (further described below), and companies bringing veterinary AI products to market are not required to report validity and performance data. A clinician may be unable to assess how an algorithm is reaching conclusions in the classic sense of the “black box”, but they may also be unable to assess performance for the clinical question at hand if this information has not been made public or assessed by the AI developer. Another downside of “black box” technology is that it prevents a clinician from learning and augmenting their practice and interpretation based on AI decisions. Insights may be gained from AI that can be used subsequently in non‐automated or “AI‐free” practice. For example, AI may correlate what a human might view as seemingly disparate variables (e.g. Alkaline phosphatase with a lung pattern) to achieve a more accurate diagnosis, which could be leveraged for improved human performance and understanding. It is not sufficient that a product can distinguish normal from pathology, we should strive to know why/how it is distinguishing normal from pathology. This must be balanced while still leveraging the power of deep learning and AI, namely to analyze and create associations the human brain cannot.^15^

Ethical considerations of datasets used in AI development include where and how datasets were sourced for algorithm training, and whether there was consent (owner, clinician/facility generating the images) for use. For a product to be considered ethical, it should have been stringently tested and validated. In order for that to occur, robust and properly curated training and validation datasets must be employed. Imaging datasets could range from combinations of images alone, images keyworded to diagnosis, images with domain expert (i.e., radiologist) report, images with consensus opinion of multiple domain experts, and presence or absence of relevant historic, clinical, biochemical, or cytological/histolpathologic information. Different datasets will create fundamentally different AI product performance and outcome. Better or worse datasets will depend on intended use to some regard, but image interpretation and accuracy by domain experts is optimized in conjunction with relevant clinical data, second opinions, and follow‐up. It stands to reason that more complete datasets would be BP for AI development vs. images alone, and engender greatest confidence in use once validated.^3^ Transparency of datasets used allows the clinician to understand whether employing that product for clinical decision‐making in real patients is ethical. It should be considered unethical to use the same data set for both training, validation, and testing purposes. If this is not disclosed to end users, a fair appraisal of the product cannot be made. How data sets were preprocessed, labeled, and validated is essential to understanding whether the resultant product is doing what is purported to do, and ultimately whether its clinical use is ethical.^18^

Ethical AI development and deployment should include disclosure of performance data, such as accuracy, sensitivity, specificity, positive/negative predictive values, scenarios/diagnoses for which the AI has been applied, and based on what datasets. If no standardized performance has been assessed this should be disclosed. It is important for the end‐user to know whether the algorithm can perform with optimal or poor‐quality imaging quality/positioning. Similarly, what methods (if any) are being employed for ongoing calibration of a released product and what (if any) performance metrics are released should be transparent and available. If no such protocols are in place this should be disclosed. Similarly, peer‐reviewed publications are an important aspect of validity with respect to algorithm performance, as the rigors of peer review apply scrutiny to claims made about an AI product's proposed utility. While lack of these components does not necessitate a product or its use to be unethical, it should raise serious questions as to why a product was released in the absence of generally acceptable scientific validation and monitoring.

Bias within datasets and resultant algorithm creation is an important topic in human medicine. Skewed data sets such as those based primarily on white males, may not be widely applicable to diagnosis in a diverse population of other sexes and races .^3^, ^15^ While we don't have the exact same considerations in veterinary medicine, inherent bias can be present in data sets. This could result from what patient populations (and the financial status of their owners) end up having imaging studies performed, whether imaging occurred at first opinion or referral practices, image quality, and if certain breeds or body types (e.g. chondrodystrophic vs. non‐chondrodystrophic) are over or underrepresented. These factors may not be evident unless the composition of the training and validation sets are known, and errors are being monitored and assessed, particularly when algorithms are not performing as expected.

### 2.3 Use

There are many potential applications of AI in veterinary medicine, and in particular veterinary imaging and radiation therapy. Ethical application of AI is inherently tied to the question of what is BP. Particularly in a landscape without a regulatory framework, how AI is deployed is an ethical question. In the absence of a regulatory framework to safeguard clinicians, patients, and their owners, veterinary medicine becomes reliant on the ethics of the developers releasing products to market. Current products are focused on clinical diagnosis or identification of pathology in images. The advantage of AI is that it is not subject to fatigue or human cognitive errors. However, as they are currently marketed and used they may serve to exacerbate cognitive errors in the humans using their output to make a clinical decision. One such error is automation bias, or the choice to accept a machine‐generated decision regardless of the clinical picture or discordant information.^19^ This leads to both errors of omission as well as commission, and is likely to be worse in the absence of a domain expert in the loop.^9^ Leveraging AI to increase domain expert access to patients would seem the most ethical path forward, whilst reducing errors such as automation bias. Image assessment is just one way that AI could facilitate a radiologist being a radiologist. Numerous other uses could increase radiologist workflow and efficiency, which has the potential to drive down cost per read and increase what a radiologist can do. These include:

- Assessment of technique and positioning at the point of care/acquisition to reduce unnecessary image number submission, and provide standardized diagnostic images of areas of interest to the interpreter.
- Worklist stratification, identifying and flagging STAT vs. non‐STAT cases.
- Optimization of hanging protocols.
- Image optimization to increase signal to noise even in suboptimal raw data.
- Natural language processing to pre‐populate reports in the radiologist's own style.
- Construction of differential diagnoses based on the radiologist's description or conclusions.
- Provision of relevant recommendations and related articles.
- Mitigate intra‐ or inter‐observer variability within or across time points, and at times when errors may be higher due to reader fatigue.

These applications do not include image diagnosis per se, but serve to off‐load time‐consuming tasks away from the radiologist, increasing productivity and accuracy, and ultimately allowing a radiologist to focus on employing their expertise.^3^, ^15^, ^20^, ^21^ Leveraging AI in this fashion (as opposed to using AI to replace radiologists) is a practical way to increase clinician and patient access to domain experts in a radiologist‐deficient market. This also provides an expert in‐line with the process, who can aid in product/algorithm optimization, error identification, and feedback as to what other applications would help domain experts perform their job better and more efficiently. These concepts are supported by a recent survey of 88 non‐radiologist clinicians across multiple specialties study in human medicine, where respondents were significantly less comfortable acting upon reports generated by AI alone versus a radiologist's report, but had similar comfort between a radiologist's report and an AI/radiologist hybrid report.^22^ Nearly 90% of clinicians in this study preferred a hybrid model compared to AI‐generating reports alone. It is worth noting that these responses come from a profession focused on one species, who receive more training (even for a family practitioner), and have more validated products due to the regulatory framework. We should then ask the question, how can it be ethical to employ these products for clinical diagnosis in veterinary medicine, across multiple species, with variable image quality and positioning, in the absence of a domain expert in the loop or similar regulatory safeguards?

Identical principles apply to the use of AI in radiation oncology where automated segmentation of organs‐at‐risk and/or tumor/target volumes have the potential to increase speed/efficiency as well as the potential to introduce medical errors that adversely impact treatment. The use of AI in the dose delivery and/or quality assurance processes in radiation oncology have the potential to lead to overdoses or underdoses that could cause harm to patients and may be very difficult to identify, even upon retrospective inspection. Inaccurate dose planning and delivery not only has potential to harm an individual patient (incomplete radiation delivery, delivery to unintended tissues), but could skew patient outcomes when those individual patients are recruited into studies for tumor response.

## 3 ETHICS OF DATA MANAGEMENT, OWNERSHIP, AND CUSTODIANSHIP

### 3.1 Sources and ownership of data

Per the AVMA veterinary ethical guidelines, medical records are the property of the practice and the practice owner.^4^ Although veterinary medicine is not bound to the Health Information Portability and Accountability Act (HIPAA) which applies to human medical data, information within veterinary medical records is confidential. This leads to several important ethical questions.

1. When and how should patient images and data be shared?
2. Is it ethical to sell patient data?
3. Is anonymization of patient data necessary or sufficient to ethically share with a third party?
4. How can informed consent to share patient data for machine learning be obtained when the general public is generally uninformed about AI?
5. If owners consent to sharing of data for machine learning, should this be shared in a centralized repository of data or sold privately to the highest bidder?
6. When patient data is used to train an algorithm, who owns the trained algorithm?

### 3.2 Consent, anonymization/privacy, and data management

It is useful to consider how other types of data are treated with respect to consent, ownership, and use. Under the EU General Data Protection Regulation (GDPR), patients own and control their data and must give explicit consent for data re‐use or sharing.^23^ Under these rules, patients must give consent to have imaging studies used to train an AI algorithm. That consent may need to be re‐obtained for each version of the algorithm.

Anonymization may be less important in veterinary vs. human medicine, but it should be recognized that real anonymization is more complicated that most people realize. Fully anonymized data sets may be manipulated in ways that allow their source to be identified. DICOM header data typically contains identifiers that include information about a patient, client, and institution. In addition, metadata is information that explains other data without its content but may also convey private information. Given that large data companies control social media platforms and AI applications, it is feasible for an entity to match veterinary patient data to human families, leading to unwanted consequences. Ethical use of patient data demands that those contributing data and AI developers be aware of these risks and take all steps possible to protect privacy such as removing DICOM header data.

While in an ideal world, all data and algorithms would be open for the public to examine, there are legitimate issues relating to cybersecurity and to protection of intellectual property and investment. Cybersecurity of data cannot be guaranteed, and a breach could result in unauthorized access to, or disclosure of personal information. Without adequate anonymization of data and informed consent, clients may not know who their data is being shared with and what additional cybersecurity risks they may be exposed to. The likelihood of consequential harm is significantly less in veterinary medicine than human medicine as it is unlikely that release of a pet's medical data would result in discrimination, humiliation, or increased insurance costs. Yet, these are not impossibilities. Any privacy breach violates the duty of veterinary providers to their clients and patients and may result in unintended harm or embarrassment.

Most risks associated with misuse of data can be at least partially managed by obtaining appropriate consent. Under the EU's GDPR, patients may give “broad consent” to have their data used for scientific research keeping within recognized ethical standards.

### 3.3 Data value and ownership

Data ownership has important implications when that data is used to generate a profitable AI product or business. It has been argued that the patients whose data are used to develop the tool should share in the resulting profits, but no mechanisms or rules are in place to guide this.^3^ There is a significant potential conflict of interests between healthcare providers, veterinary clients/patients, AI developers, and the overarching public interest in open access to data that can improve medical care.^3^ An acceptable principle is that a patient can only be considered to have given consent for others to use their data if they have also been informed of the monetary value of that data.^3^, ^24^ Unfortunately, intellectual property rights for work derived from patient health data is ambiguous.

### 3.4 Data sharing and custodianship

When consent has not been specifically granted for a specific purpose/project, the data custodian (veterinary hospital, radiologist) acts as the gatekeeper for access to that data. In that role, they are in a position to determine what projects can use the data and to some degree how the data is used, in conjunction with I.A.C.U.C. or research ethical review boards. As data becomes an important commodity, to what extent should a data custodian be able to charge for granting a third‐party access to de‐identified patient data? Furthermore, should pet owners share in profits generated from their pet's medical data? Guidelines must be established as to what is BP for data sharing and custodianship.

## 4 RESPONSIBILITY AND LIABILITY

### 4.1 AI within the VCPR and liability

When considering the use of AI, we must consider who or what is liable in the case of a negative outcome. Is liability held by the veterinarian using the AI, the hospital who employs it, or the AI developer?^15^ In order to approach these questions, we must first define the veterinary‐client‐patient relationship (VCPR) and who holds it. In order for a VCPR to be established, several criteria must be met.

1. A veterinarian must have assumed responsibility for clinical decisions regarding patient health with client consent.
2. That veterinarian must have adequate patient knowledge to initiate a preliminary diagnosis on the patient's condition, including timely examination of the patient and knowledge of how the patient is kept and cared for.
3. The veterinarian should be available for follow‐up assessment or arrange for continued care.
4. The veterinarian oversees treatment, compliance, and outcome.
5. The veterinarian must maintain patient records.^25^

In the current setting of AI use, the receiving veterinarian (a non‐radiologist, and often a non‐specialist veterinarian) holds the VCPR, and as a result the liability as they will decide upon diagnostic and treatment choices. According the American Veterinary Medical Association (AVMA) Principles of Veterinary Medical Ethics*, “*When appropriate, attending veterinarians are encouraged to seek assistance in the form of consultations and/or referrals. A decision to consult or refer is made jointly by the attending veterinarian and the client. When a private clinical consultation occurs, the attending veterinarian continues to be primarily responsible for the case and maintaining the VCPR. Consultations usually involve the exchange of information or interpretation of test results. However, it may be appropriate or necessary for consultants to examine patients. When advanced or invasive techniques are required to gather information or substantiate diagnoses, attending veterinarians may refer the patients. A new VCPR is established with the veterinarian to whom a case is referred.^25^” We should then consider exactly what AI decision‐making is within these guidelines. Is this a telemedicine referral with a non‐human colleague? Is this a consult? Is this simply a diagnostic akin to running a blood chemistry? If this is a consult or referral, it would seem that owner consent to use of AI in decision making is required and should be disclosed and sought prior to use. The current paradigm certainly involves the exchange of information and interpretation of imaging by AI, which sounds a lot like a consult. This again begs the question of whether AI does, should, or even can hold the VCPR in these interactions. Also, within the AVMA Principles is, “Referral is the transfer of responsibility of diagnosis and treatment from a referring veterinarian to a receiving veterinarian.^25^” While this would seemingly not include an AI product or company as constituting referral from the standpoint of AI not being a veterinarian, it could be considered a referral if responsibility of diagnosis and treatment is shifted to the AI. We must also acknowledge that these principles were created at a time when the concept of AI producing a diagnosis was not yet a reality. If AI is performing tasks similar to what a receiving consulting veterinarian would, it begs the question whether a new VCPR is or should be established by the AI/developer. If so, then there should be resultant shifting of liability away from the receiving veterinarian to the AI/developer in instances of their use. This is particularly relevant in the current landscape where the receiving veterinarian holds the VCPR and resultant liability, but may not be provided with appropriate information or have the ability to assess the validity of an AI product nor the diagnoses and recommendations it produces. It becomes increasingly clear that these are issues that need to be addressed proactively, ideally prior to release of these products into the market. The lack of intentional planning on these issues runs the risk of it being brought to task by an index case guided by evolving tort law, with the untoward effect of not only individual patient, client, or veterinarian harm, but stifling of continued use and advancement of these new technologies.

Harm from AI could be caused in many ways. An AI product could be non‐contributory to patient diagnosis/care, merely adding to client costs. It could yield a false‐positive diagnosis, leading to subsequent tests or interventions (up to and including surgery or euthanasia). It could lead to false‐negative results, delaying necessary diagnosis and care. It could be applied to inappropriate datasets or populations (e.g. applying an algorithm to a non‐diagnostic image with questionable results). It could be employed incorrectly by a human user. The AI could simply be faulty and generate erroneous decisions. Incorrect results could lead to subsequent faulty data in research projects sampling this data. In human medicine, the Canadian Association of Radiologists have produced a white paper on this topic, with a proposed scheme to address who holds the responsibility/liability in the instance of misdiagnosis or malpractice when AI is employed (Table 1). A version of this could certainly be adopted in veterinary medicine.

**TABLE 1:** Levels of automation of AI systems in veterinary radiology^26^

Adapted from CAR Level of Automation for AI Systems			
ACVR Level	Name	Description	Liability
0	No automation	Interpretation/intervention performed solely by veterinarian (ideally radiologist)	Veterinarian/Radiologist
1	Veterinarian assistance	Interpretation/intervention performed primarily by veterinarian (ideally radiologist) with secondary oversight by AI (e.g. worklist prioritization).	Veterinarian/Radiologist
2	Partial automation	Interpretation/intervention performed primarily by AI with secondary oversight by veterinarian (ideally radiologist) (e.g. report pre‐population, pathology detection).	Veterinarian/Radiologist
3	Conditional automation	Interpretation/intervention performed solely by AI for a specific indication. The veterinarian (ideally radiologist) intervenes if results are positive or inconclusive (e.g. AI triages a case and identifies tension pneumothorax and is reviewed by veterinarian, but an AI triaged normal study is not reviewed).	AI/ Veterinarian/Radiologist
4	High automation	Interpretation/intervention performed solely by AI for a specific indication without expectation of review by a veterinarian (ideally radiologist). AI generates diagnosis and recommendations autonomously (e.g. AI identifies aggressive bone lesion on radiographs and recommends biopsy and staging).	AI
5	Full automation	Interpretation/intervention performed solely by AI for all indications expected of a veterinarian (radiologist). AI generates diagnosis(es) and recommend management autonomously (e.g. thoracic radiographs are performed for a patient with an anal sac tumor to check for metastases, AI reports no metastases but identifies pneumonia and an aggressive bone lesion on edge of radiograph and recommends further treatment and imaging).	AI

It is important to highlight that in human medicine, the proposed liability scheme is based on radiologists/domain expert as the human in the loop, which is not the current paradigm in veterinary medicine. Despite this being a more robust system in human medicine, when surveyed, non‐radiologist clinicians in human medicine felt that liability for errors when using AI should fall on the hospital (65.9%), the radiologist (54.5%), and the AI developer (44.3%). Only 4.5% felt that liability should be held by the referring physician.^22^ If our human colleagues are not comfortable using AI reports alone from products which are more rigorously tested, why should veterinarians (in particular non‐domain experts) feel comfortable using products with no oversight? We must address who/what holds the VCPR, what constitutes a referral, and who holds the resultant liability when AI is used. In particular, so our non‐domain expert colleagues are not left holding the liability bag. We should ask ourselves as a profession, is it ethical to shift liability to general practitioners, emergency veterinarians, or non‐imaging specialists who use a product whose accuracy is not published or otherwise known? Is the average veterinary clinic or hospital (which may be individually or corporate owned) prepared to accept liability of their use? If AI begins to perform similar diagnostic tasks to a veterinarian, shouldn't AI/developers also then be held to the same standards including the guiding principle of “first, do no harm”?^9^

### 4.2 Regulatory oversight

Regulation and oversight, or lack thereof, is one of the key differences when considering the use of AI in veterinary medicine versus human medicine. The regulatory framework in human medicine is actively evolving, as our human medical colleagues navigate how to best employ and safeguard these emerging technologies. In order to understand ethical implications in veterinary medicine, we must consider the current regulatory landscape. The United States Food and Drug Administration (FDA) regulates medical devices. Section 201(h) of U.S. Federal Food, Drug, and Cosmetics Act (FDCA), defines a medical device as, “as instrument, apparatus, implement, machine, contrivance, implant, in vitro agent or other similar or related article…intended for use in the diagnosis of disease or other conditions, or in the cure, mitigation, treatment, or prevention of disease, in man or other animals…intended to affect the structure or any function of the body of man or other animals”.^12^ AI and similar products have come to be regarded as software as a medical device (SaMD).^26^, ^27^ However, the 21^st^ Century Cures Act (Pub. L. No. 114–255) introduced an exemption in section 520(o) of the FDCA for some software. In order to be exempt from software being considered a medical device, it must be used for, “…administrative support…”, “…to serve as electronic patient records and is not intended to interpret or analyze patient records…”, “… transferring, storing, converting formats, or displaying clinical laboratory test or other device data and results…”, and “…for maintaining or encouraging a healthy lifestyle and is unrelated to diagnosis, cure, mitigation, prevention, or treatment of a disease or condition”.^27^ Section 520(o)(1)(E) further states that to be exempt software must meet several criteria simultaneously, including, “…not intended to acquire, process, or analyze a medical image or signal from an in vitro diagnostic device or a pattern or signal from a signal acquisition system…”, “…for the purpose of… supporting or providing recommendations to a healthcare professional about prevention, diagnosis, or treatment of a disease or condition”, and is, “…for the purpose of…enabling such healthcare professionals to independently review the basis for such recommendations that such software presents so that is not the intent that such healthcare professionals rely primarily on any of such recommendations to make a clinical diagnosis or treatment decision regarding an individual patient”.^27^ These guidelines clearly support the radiologist in the loop concept, and that AI products used to generate diagnoses would be classified as medical devices (and subject to their regulation). The FDA has gone on to provide clarifications that echo guiding principles in AI development proposed earlier in this manuscript, advising that, “…the software developer should describe the underlying data used to develop the algorithm and should include plain language descriptions of the logic or rationale used by an algorithm to render a recommendation”, and that, “…the sources supporting the recommendation or the sources underlying the basis for the recommendation should be identified and available to the intended user”. Transparency of data sets, avoidance of black box development, and access to development information are clear guidelines from the FDA. In order to foster innovation, the FDA also offers that it, “…does not intend to enforce compliance even if the last criterion is not met (decisions primarily on software recommendations) as long as it is used for non‐serious or non‐critical conditions”.^27^ This last statement has led to the creation of a risk stratification framework for the use of AI. Adapted versions of these frameworks are provided in Tables 2 and 3.

**TABLE 2:** Framework for risk categorization from the International Medical Device Regulators^27^, ^29^

**Adapted from the International Medical Device Regulators Framework**			
**Category of Condition**	**Information Provided by SaMD/AI to Healthcare Decision**		
	**Treat or Diagnose**	**Drive Clinical Management**	**Inform Clinical Management**
**Critical**	IV	III	II
**Serious**	III	II	I
**Non‐serious**	II	I	I

**TABLE 3:** Framework for risk categorization from the EU Medical Device Regulators^30^

**Adapted from E.U. Medical Device Regulation**				
**Class**	**I**	**IIa**	**IIb**	**III**
**Risk Level**	**Low**	**Medium**	**Higher**	**Highest**
**Description**		Decisions with diagnostic or therapeutic purposes, or intended to monitor physiologic processes.	Decisions that may cause serious deterioration of state of health or lead to surgical intervention, or if for physiologic parameters where variations could lead to immediate patient danger.	Decisions that may cause death or an irreversible deterioration of health.

Applying these frameworks to veterinary medicine, it is evident that almost every possible decision of current AI products would fall into medium risk (decisions having diagnostic or therapeutic purposes), and many would fall into higher or highest risk. Remembering the “Euthanasia Principle”, patients with AI diagnoses such as “pulmonary nodules‐metastases”, “aggressive bone lesion”, or “mechanical ileus/obstruction” may lead to euthanasia or invasive treatment depending on prognosis and client finances. As many decisions in veterinary medicine must be made at a single visit, there are very few if any AI‐assisted imaging diagnoses that are low risk. Coupled with what is often little to no follow up on patients or their diagnoses, there are less checks and balances to catch an incorrect AI diagnosis vs. human medicine. Applying current FDA guidelines for human AI products, AI as it is currently being employed in veterinary medicine should be considered a medical device/SaMD and subject to appropriate testing, oversight, and regulation. The lack of such regulation is another key difference between human and veterinary medicine. The FDA currently has no requirements for pre‐market approval of medical devices intended for animal use.^28^ This means there are no restrictions to bringing an AI product to the veterinary market, and no safeguards to ensure proper testing, accuracy, or performance. This begs some key questions. In the absence of any governmental regulatory framework to safeguard veterinary AI products, can we rely on AI developers alone to properly test and ethically deploy these products? How can an end user properly evaluate AI products for themselves in the absence of necessary data? Is it ethical to test AI products on clinical patients without consent? How and to what extent should these considerations be disclosed to owners when AI products are used in the diagnosis of their non‐human family? How can risk be properly disclosed if this information is not known by the end‐user veterinarian or provided by the AI developer?

Barring large‐scale legislative changes to safeguard patients and their owners, it becomes the responsibility of the profession to safeguard our patients, our clients, and ourselves from potentially harmful AI products. This should be led by domain experts, namely the ACVR/ECVDI and their constituency, at least in how it relates to diagnostic imaging and radiation oncology. Guidelines must be developed for what to look for in a validated AI product, how to identify questionable products, how to assess performance, error reporting, and future ethical development. While the ACVR/ECVDI are not regulatory bodies, criteria could be developed which AI developers could meet, similar to AAFCO statements in veterinary nutrition. This would at least provide some assurance that a product has met minimum requirements of what domain experts deem sufficient for preliminary use. Against the backdrop of otherwise absent regulations, it further supports the necessity of the radiologist in the loop when AI products are employed in live patients for the foreseeable future. AI developers with ethical aims should seek involvement and adoption by domain experts as the BP path forward.

In human medicine, this is further regulated whether an AI is “locked” once released to market (i.e.; the algorithm does not change over time), versus additional regulatory approvals when substantive changes to the AI are made.^27^ The advantage of AI is the ability for it to continuously learn and improve over time. With no oversight how is the end user to know if an AI is performing better or worse than prior iterations, or if it is being improved iteratively at all? This question again highlights the importance of transparency from AI developers, so that end‐users can be conscientious in the use of AI.

### 4.3 Ethical marketing of AI

With no regulatory safeguards or hurdles preventing entry of AI products into the veterinary marketplace, their adoption and use or lack thereof will largely become a function of product marketing and first‐hand use. In the AVMA Principles of veterinary medical ethics, “Advertising by veterinarians is ethical when there are no false, deceptive, or misleading statements or claims. A false, deceptive, or misleading statement or claim is one which communicates false information or is intended, through a material omission, to leave a false impression.^4^” This is further echoed in FDA guidance where an animal medical device is considered misbranded if, “labeling is false or misleading”.^28^ Prevention of inappropriately tested products from entering the market may not be feasible under the current regulatory landscape, but the profession and domain experts in particular can play a role in alerting proper authorities when these codes of ethical marketing have been breached. Ultimately, this will have to be met with educational marketing from domain experts (radiologists and radiation oncologists), to inform non‐domain experts of what to look for in trustworthy or questionable products, what we feel proper and ethical use for these technologies are, and which if any products we endorse as constituents of the ACVR/ECVDI.

## 5 GENERAL RECOMMENDATIONS FOR ETHICAL USE OF AI IN VETERINARY MEDICINE

- AI technology does no harm.
- Radiologists and other domain experts should be “in the loop” from start to finish of development, deployment, and supervision of AI products.
- AI companies and their products should be transparent, and provide/disclose information relating to data use, validation and training, calibration, outcomes, and errors.
- AI products should be subject to peer review (ideally prior to entry into the market for clinical use) and guided by position/white papers by domain experts (e.g. ACVR/ECVDI) when available.
- When medical errors occur, a root‐cause analysis should be performed to identify to points at which decision making was faulty. Ideally this would be shared on a national database. Companies should be transparent when errors occur.
- Until further progress is made, the profession should strive to have radiologists involved in final imaging diagnosis in conjunction with AI, rather than by AI alone.

## CONFLICT OF INTEREST

The authors have declared no conflict of interest.

## LIST OF AUTHOR CONTRIBUTIONS

Category 1

- Conception and Design: Cohen, Gordon
- Analysis of Data: Cohen, Gordon
- Interpretation of Data: Cohen, Gordon

Category 2

- Drafting the Article: Cohen, Gordon
- Revising It Critically for Important Intellectual Content: Cohen, Gordon

Category 3

- Final Approval of the Version to be Published: Cohen, Gordon

Category 4

- Agreement to be accountable for all aspects of the work in ensuring that questions related to the accuracy or integrity of any part of the work are appropriately investigated and resolved: Cohen, Gordon
